# Culturable Opportunistic Bacteria and Antimicrobial Susceptibility Patterns in Canine Periodontal Pockets: A Pilot Study

**DOI:** 10.3390/ani16162490

**Published:** 2026-08-11

**Authors:** Laura Šakarnytė, Lina Merkevičienė, Rita Šiugždinienė, Modestas Ružauskas

**Affiliations:** 1 Microbiology and Virology Institute, Lithuanian University of Health Sciences, 44307 Kaunas, Lithuaniamodestas.ruzauskas@lsmu.lt (M.R.); 2 Department of Anatomy and Physiology, Veterinary Academy, Lithuanian University of Health Sciences, 44307 Kaunas, Lithuania

**Keywords:** canine periodontitis, periodontal pockets, *Pasteurella stomatis*, *Neisseria animaloris*, *Streptococcus canis*, MIC, antimicrobial susceptibility, One Health

## Abstract

Canine periodontitis is one of the most prevalent chronic diseases for dogs over two years of age. Periodontal pockets may serve as a reservoir for opportunistic bacteria including *Pasteurella stomatis*, *Neisseria animaloris*, and *Streptococcus canis*; however, data on their antimicrobial susceptibility and potential One Health relevance remain limited. This pilot study investigated culturable bacterial isolates recovered from periodontal pockets of dogs with different stages of periodontal disease. Subgingival samples were collected under general anesthesia after periodontal probing and dental radiographic assessment during routine comprehensive oral health assessment and treatment (COHAT) procedures. Bacterial isolates were cultured, identified using MALDI-TOF MS, and tested for minimum inhibitory concentrations (MIC) against amoxicillin (AML), doxycycline (DX), trimethoprim–sulfamethoxazole (SXT), gentamicin (CN), chloramphenicol (C), and ciprofloxacin (CIP). In total, 122 isolates from 101 dogs were analyzed, including *Pasteurella stomatis* (n = 54), *Neisseria animaloris* (n = 33), and *Streptococcus canis* (n = 35). MIC distributions varied between bacterial species and antimicrobial agents, while interpretation was limited by the lack of species-specific veterinary medicine breakpoints for some isolates. These preliminary findings highlight the need for larger standardized studies to support antimicrobial resistance surveillance in canine oral microbiota.

## Simple Summary

Periodontal disease is very common in dogs and can create pockets around the teeth where bacteria accumulate. This study looked at three zoonotic species of bacteria that can live in these pockets—*Pasteurella stomatis*, *Neisseria animaloris,* and *Streptococcus canis*. Samples from 101 dogs with different degrees of gum disease were collected and investigated. In total, 122 bacterial isolates were obtained and tested for antimicrobial susceptibility. The results obtained demonstrated increased carriage of bacterial species in dogs with advanced gum disease. Antimicrobial resistance to important classes of antibiotics for animals and humans was detected, highlighting not only the risk associated with bite wounds but also the importance of hygiene when interacting with one’s pets.

## 1. Introduction

Periodontitis is one of the most common chronic diseases in dogs, affecting over 80% of dogs by three years of age, and is characterized by inflammation of gingiva, periodontal tissues, and bone loss [1]. This disease begins with plaque accumulation and gingivitis, and may progress to periodontitis with periodontal pocket formation, attachment loss, and tooth mobility [2]. Its occurrence is influenced by several factors, including age, breed, oral hygiene, diet, and local anatomical conditions [3]. The pathogenesis of periodontitis is closely associated with dental plaque biofilms forming bacteria. Biofilms provide a structured microbial environment in which bacteria are protected from host defenses and allow complex interactions between microbial communities and the host immune response [4].

While the pathogenesis of periodontitis is increasingly well characterized, the specific bacterial species colonizing periodontal pockets remain underexplored. Previous studies have shown that the canine oral microbiome is highly diverse and differs substantially from the human oral microbiome [5,6]. Among culturable bacteria recovered from canine periodontal pockets, *Pasteurella* spp., *Neisseria* spp., and *Streptococcus* spp. are particularly relevant and may occur as commensal microorganisms of the oral and upper respiratory tract of dogs but can also be opportunistic pathogens and cause zoonotic diseases [7,8,9,10]. *Pasteurella stomatis* is a Gram-negative, facultatively anaerobic member of the *Pasteurellaceae* family and is considered part of the oral and upper respiratory microbiota of domestic carnivores. Its biological relevance increases when the oral mucosal barrier is disrupted. Reported infections range from mild skin lesions to severe systemic disease, highlighting its relevance [10,11]. *Neisseria animaloris* is a Gram-negative bacterium mainly linked to dogs’ and cats’ oral and upper respiratory microbiota but can also cause opportunistic wound infections after bites or close animal contact with humans [8,12]. *Streptococcus canis* is a β-haemolytic Lancefield group G streptococcus commonly associated with domesticated animals like dogs and cats and has likewise been associated with opportunistic infections in animals and humans [9,13]. The broader epidemiology is poorly understood, which highlights the need for further evidence. Despite the clinical relevance of canine oral opportunists such as *Pasteurella* spp., *Neisseria* spp. and *Streptococcus* spp., antimicrobial resistance data remain limited, particularly for isolates recovered from periodontal pockets. Interpretation of MIC values is further complicated by the absence of species-specific veterinary clinical breakpoints for several animal-associated oral bacteria. This limitation is important because reliable MIC breakpoints require substantial data that are insufficient for many pathogens [13,14].

Addressing this gap is not merely of academic interest. Surveillance of MIC distributions in canine periodontal isolates is valuable for identifying potential reservoirs of resistant bacteria at the human–animal interface, providing a logical basis for considering antimicrobial resistance from a One Health perspective [15,16]. Given the close and frequent contact between dogs and their owners, periodontal pockets harboring opportunistic, potentially resistant bacteria represent a plausible, yet underexamined, point of convergence between companion-animal and human health. In this study, we therefore investigated the presence and antimicrobial susceptibility of culturable *P. stomatis*, *N. animaloris,* and *S. canis* isolates recovered from the periodontal pockets of dogs with varying degrees of periodontal disease. As a pilot study, our analysis of 101 dogs and 122 isolates provides preliminary evidence rather than definitive conclusions. Future studies involving larger, standardized cohorts and expanded antimicrobial panels will be essential to support breakpoint development and strengthen antimicrobial resistance surveillance efforts.

## 2. Materials and Methods

### 2.1. Study Design and Scoring Criteria

**Figure 1:**
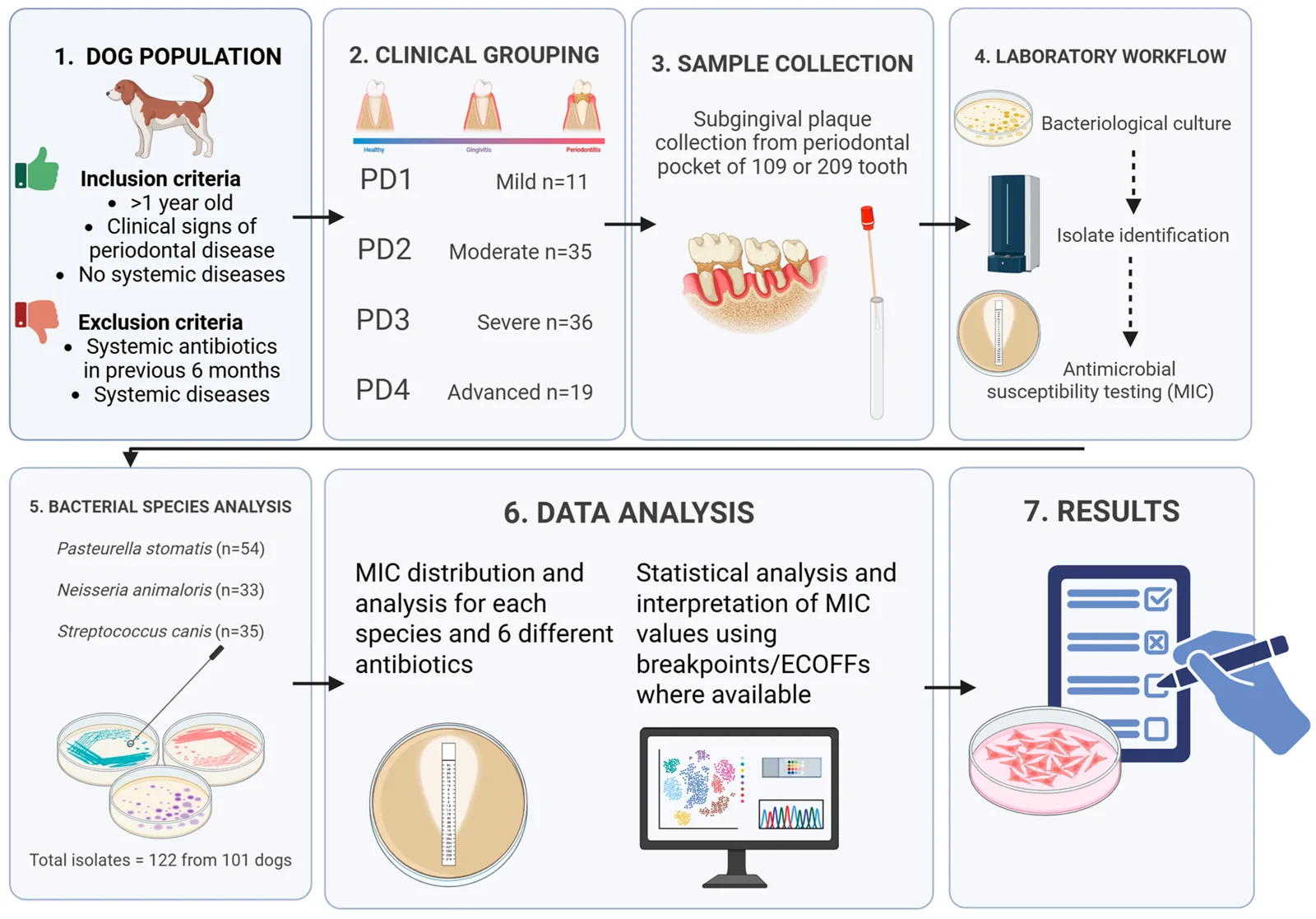
Schematic representation of the study design and workflow.

This study included 101 client-owned dogs presented to a veterinary hospital in central Lithuania. Prior to enrolment, written informed consent was obtained from each owner. All procedures were performed in accordance with applicable international, national, and institutional guidelines for animal care and use. Ethical approval was obtained from the Bioethics Center of the Lithuanian University of Health Sciences, Kaunas, Lithuania, approval number 2024-BEC3-T-005; all dog owners signed a consent form acknowledging that sample collection was an ancillary part of the procedure, not its primary purpose. Dogs were included if they were at least 1 year of age, had no evidence of systemic disease, and had not received systemic antibiotics during the previous 6 months. General health status was assessed by standard veterinary examination, including morphological and biochemical blood analyses. Oral examinations were performed by a certified veterinary odontologist under general anesthesia using an inhalant anesthesia protocol during routine dental procedures at the veterinary hospital. Dental radiography and periodontal probing were performed to support periodontitis diagnosis and disease staging. Figure 1 summarizes the overall study workflow, including dog selection, clinical grouping according to periodontal disease severity, bacterial culture and species identification, antimicrobial susceptibility testing, and final analysis.

**Figure 2:**
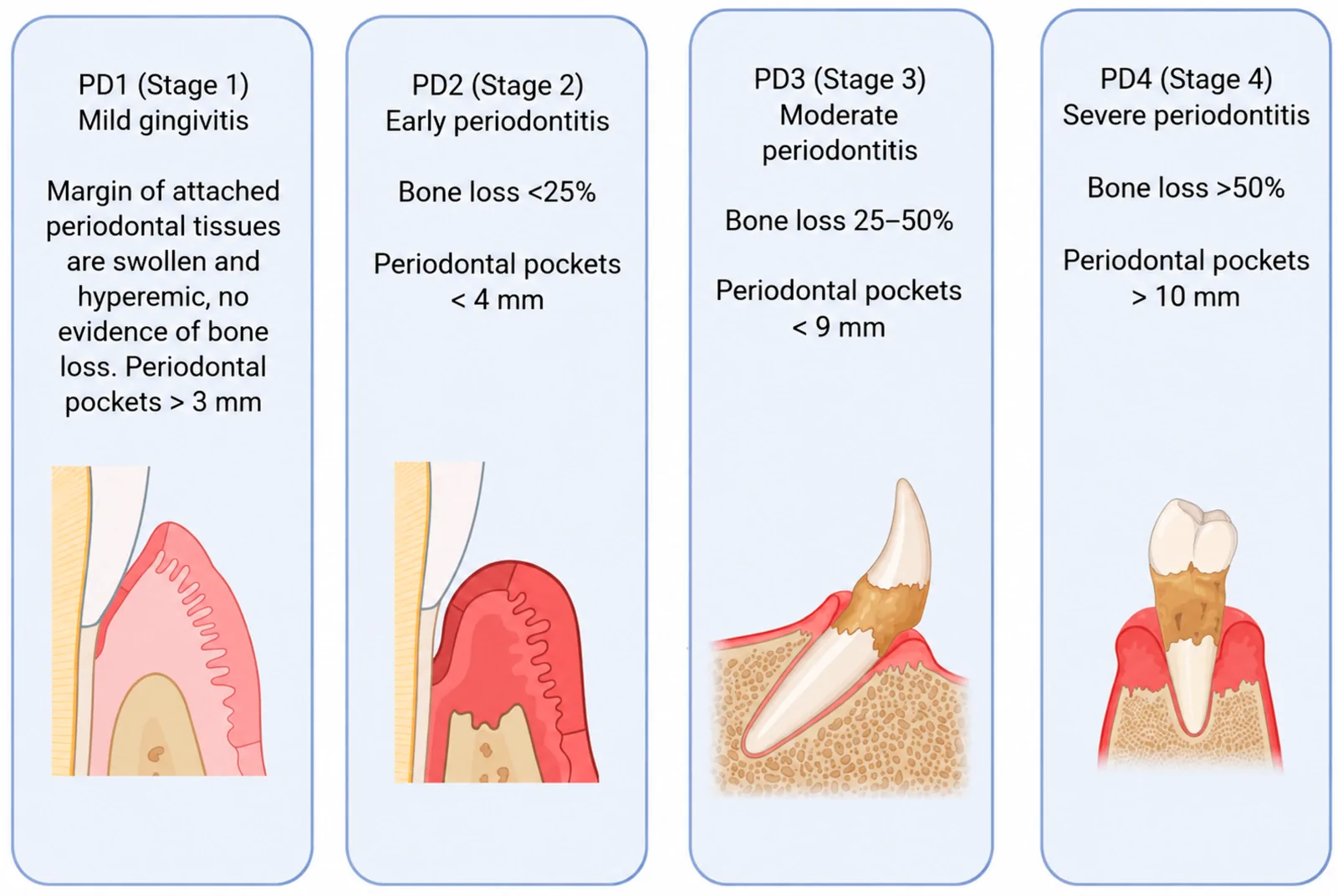
Periodontal disease scoring criteria used for clinical grouping of dogs, PD—periodontal disease.

Dogs were classified into four periodontal disease groups according to clinical and radiographic findings, as shown in Figure 2. PD1—mild periodontal disease (n = 11): mild gingivitis with swollen and hyperemic attached periodontal tissues with no radiographic evidence of bone loss. PD2—moderate periodontal disease (n = 35): periodontal pockets smaller than 4 mm and bone loss less than 25%. PD3—moderate periodontal disease (n = 36): 25–50% bone loss and periodontal pockets 4–9 mm. PD4—severe periodontal disease (n = 19): bone loss greater than 50% and periodontal pockets deeper than 10 mm.

### 2.2. Collection of Samples

After each dog was placed under general anesthesia, periodontal probing and evaluation were performed, including dental radiography examinations, to assess the severity of periodontal disease. Subgingival samples were collected from an affected tooth (109 or 209) periodontal pocket, depending on the localization and severity of periodontal lesions. Samples were obtained using sterile Transwab^®^ swabs (Medical Wire and Equipment Co., Ltd., Corsham, UK). After collection, the swabs were immediately placed in a refrigerator and stored at 4 °C. All samples were transported to the laboratory within 12 h of collection, maintaining a temperature of 4 °C during transportation.

### 2.3. Classical Bacteriological Culture Analysis and Mass Spectrometry Identification

Samples collected from periodontal pockets were plated onto Columbia agar supplemented with 5% horse blood and fastidious anaerobe agar supplemented with 5% horse blood (Liofilchem, Roseto degli Abruzzi, Teramo, Italy). The inoculated plates were incubated at 37 °C under both aerobic and anaerobic conditions for up to 72 h. After incubation, bacterial growth was evaluated based on colony morphology. To obtain pure cultures, up to four of the most prevalent but morphologically distinct colony types for each sample were selected and subcultured onto fresh agar plates [17]. Pure isolates were used for further bacterial species identification and antimicrobial susceptibility testing.

Bacterial isolates were identified using matrix-assisted laser desorption/ionization time-of-flight mass spectrometry (MALDI-TOF MS) with the MALDI Biotyper Sirius system (Bruker Daltonics GmbH, Bremen, Germany) at the Institute of Microbiology and Virology, Lithuanian University of Health Sciences, Kaunas, Lithuania. The generated protein spectra were compared in real time with the MALDI Biotyper reference database, library version 2023 [18], containing spectra from a broad range of microorganisms. Identification reliability was interpreted according to the manufacturer’s scoring criteria: scores of ≥2.0 were considered reliable for species-level identification, while scores between 1.7 and 2.0 were accepted for genus-level identification [19].

### 2.4. Antibiotic Resistance Testing

Antimicrobial susceptibility testing was performed for identified *Pasteurella stomatis*, *Neisseria animaloris*, and *Streptococcus canis* isolates. Minimum inhibitory concentrations (MICs) were determined using MIC test strips (Liofilchem, Italy) on 140 mm Mueller–Hinton Fastidious Agar supplemented with 5% horse blood and 20 mg/L β-NAD (Liofilchem, Italy). The following antimicrobials were tested: amoxicillin, doxycycline, trimethoprim–sulfamethoxazole, gentamicin, chloramphenicol, and ciprofloxacin. MIC results were interpreted according to available clinical breakpoints recommended by the European Committee on Antimicrobial Susceptibility Testing (EUCAST) (whenever possible) [20] and the Clinical and Laboratory Standards Institute (CLSI) [21], and epidemiological cut-off values (ECOFFs) were analyzed [22]. As there are no clinical breakpoints set for ciprofloxacin against *Streptococcus* spp. in EUCAST and CLSI guidelines, the clinical breakpoint for ciprofloxacin was used from the standards set by the US Food and Drug Administration (FDA) for beta-hemolytic streptococci of human clinical origin, as this breakpoint has not been specifically validated for *Streptococcus canis* [23].

### 2.5. Data Processing and Analysis

Data were processed and analyzed using Microsoft Excel 2019. Dogs were grouped according to periodontal disease severity, and bacterial species and antimicrobial susceptibility results were summarized for each group. The frequencies of bacterial isolates and resistant strains were calculated as counts and percentages. Schematic figures were made using www.biorender.com.

## 3. Results

### 3.1. Distribution of Bacterial Isolates

In total, 122 bacterial isolates were obtained and included in the analysis: 54 *Pasteurella stomatis*, 33 *Neisseria animaloris*, and 35 *Streptococcus canis*—these isolates were recovered from 101 unique dogs (Table 1). Each isolate was analyzed in 2–3 replicate spots on the target plate, and the identification result with the highest score was recorded for species-level assignment; all isolates were identified with scores ≥2.0. Among the 101 dogs positive for at least one of the three bacterial species investigated, 50.49% (51 dogs) harbored one species, 35.64% (36 dogs) harbored two species, and 13.86% (14 dogs) harbored all three species. Based on the periodontal disease score recorded for each dog, 11 dogs were assigned to periodontal disease stage 1 (PD1), 35 to stage 2 (PD2), 36 to stage 3 (PD3), and 19 to stage 4 (PD4) (Table 2).

### 3.2. Dataset Characteristics and Composition of Bacterial Isolates

#### 3.2.1. *Pasteurella stomatis*

The distribution of *P. stomatis* isolates according to antibiotic MIC values is summarized in Table 3. A total of 54 isolates were included. Gentamicin MICs were mainly distributed around 1.5 mg/L (9/54, 16.7%), 0.19 mg/L (7/54, 13.0%), 0.50 mg/L (7/54, 13.0%), and 0.75 mg/L (7/54, 13.0%). Notably, 2/54 (3.7%) isolates showed an MIC of 1024 mg/L. For doxycycline, the MIC distribution was centered mainly at 0.25 mg/L (11/54, 20.4%) and 0.19 mg/L (9/54, 16.7%), although 2/54 (3.7%) isolates showed very high MIC values of 256 mg/L. For trimethoprim–sulfamethoxazole, the most frequent MICs were 0.125 mg/L (8/54, 14.8%), 0.047 mg/L (8/54, 14.8%), and 0.064 mg/L (7/54, 13.0%), although 3/54 (5.6%) isolates reached 32 mg/L. Chloramphenicol MICs were most often 0.75 mg/L (13/54, 24.1%) and 0.38 mg/L (12/54, 22.2%), with only 1/54 (1.9%) isolate showing an MIC of 12 mg/L. Amoxicillin MICs were concentrated at 0.016 mg/L (8/54, 14.8%) and 0.094 mg/L (8/54, 14.8%), although 2/54 (3.7%) isolates had an MIC of 256 mg/L. Ciprofloxacin MICs were concentrated most commonly at 0.004 mg/L (10/54, 18.5%), 0.002 mg/L (7/54, 13.0%), and 0.006 mg/L (7/54, 13.0%), while 2/54 (3.7%) isolates had MICs of 32 mg/L. Clinical breakpoints, whenever possible for *Pasteurella* spp., were used to detect resistant isolates, whereas the rest of the data were interpreted descriptively based on MIC distribution.

#### 3.2.2. *Neisseria animaloris*

The distribution of *N. animaloris* isolates according to MIC values is presented in Table 4. A total of 33 isolates were analyzed. Gentamicin MICs clustered predominantly at 0.75 mg/L (10/33, 30.3%), followed by 0.50 mg/L (5/33, 15.2%) and 1 mg/L (4/33, 12.1%). Doxycycline MICs were most frequently observed at 0.50 mg/L (7/33, 21.2%), followed by 8 mg/L (5/33, 15.2%), while 1/33 (3.0%) isolate showed an MIC of 256 mg/L. For trimethoprim–sulfamethoxazole, the most frequent MIC was 0.25 mg/L (8/33, 24.2%), followed by 0.50 mg/L (5/33, 15.2%), 0.125 mg/L (4/33, 12.1%), and 0.38 mg/L (4/33, 12.1%); 2/33 (6.1%) isolates showed MICs of 32 mg/L. Chloramphenicol MICs were most commonly 1 mg/L (9/33, 27.3%) and 1.5 mg/L (5/33, 15.2%), whereas 2/33 (6.1%) isolates had MICs of 256 mg/L. Amoxicillin MICs were concentrated at low concentrations, particularly 0.125 mg/L (6/33, 18.2%) and 0.19 mg/L (6/33, 18.2%), while 1/33 (3.0%) isolate showed an MIC of 256 mg/L. Ciprofloxacin MICs were concentrated most commonly at 0.003 mg/L (5/33, 15.2%). As no validated species-specific clinical breakpoints or cut-off values are currently available for *N. animaloris*, the MIC data were interpreted descriptively based on their distribution.

#### 3.2.3. *Streptococcus canis*

The distribution of *S. canis* isolates according to MIC values is presented in Table 5. A total of 35 isolates were analyzed. Gentamicin MICs were most frequently observed at 6 mg/L (8/35, 22.9%), followed by 2 mg/L (6/35, 17.1%) and 4 mg/L (5/35, 14.3%). Doxycycline MICs were most commonly 6 mg/L (4/35, 11.4%) and 256 mg/L (4/35, 11.4%), while 3 mg/L, 4 mg/L, and 12 mg/L each accounted for 3/35 (8.6%) isolates. For trimethoprim–sulfamethoxazole, the most frequent MICs were 0.094 mg/L (6/35, 17.1%) and 0.125 mg/L (6/35, 17.1%), followed by 0.19 mg/L (4/35, 11.4%). Chloramphenicol MICs were most frequently observed at 12 mg/L (7/35, 20.0%), while 3 mg/L and 8 mg/L each accounted for 6/35 (17.1%), and 6 mg/L for 5/35 (14.3%) isolates. Amoxicillin MICs were concentrated mainly at 0.19 mg/L (6/35, 17.1%), followed by 0.094 mg/L (4/35, 11.4%), whereas 0 mg/L, 0.38 mg/L, 0.50 mg/L, and 256 mg/L were each observed in 3/35 (8.6%) isolates. Ciprofloxacin MICs were centered mainly at 2 mg/L (5/35, 14.3%), while 0.75 mg/L, 1 mg/L, 1.5 mg/L, 3 mg/L, and 32 mg/L each accounted for 3/35 (8.6%) isolates.

## 4. Discussion

This pilot study focused on three culturable opportunistic bacterial species from canine periodontal pockets—*Pasteurella stomatis*, *Neisseria animaloris*, and *Streptococcus canis*. It specifically targeted viable bacterial isolates that could be cultured, identified to species level and further evaluated by MIC testing. This is important because metagenomic and next-generation-sequencing studies have already shown that the canine oral microbiome is highly diverse and shows only limited overlap with the human oral microbiome—only about 5.9% of species [5,24]. Furthermore, culture-based data are valuable not only for taxonomic description but also for antimicrobial susceptibility testing, which is useful for both fundamental data and for clinicians.

A notable finding of this study is that the number of bacterial species detected per dog appeared to increase with periodontal disease severity. Dogs with early-stage periodontitis more commonly harbored only one of the investigated species, whereas dogs with more advanced periodontitis more frequently carried two or even three investigated species. Such data demonstrate possible synergistic influence of investigated pathogens not only in the etiology of the disease but also on disease severity. It is biologically plausible that greater periodontal disease creates a more permissive ecological niche for the persistence of multiple opportunistic organisms [25,26]. Similar changes in bacterial composition across different stages of canine periodontitis have been described previously, supporting the concept that periodontal disease is associated with microbial shifts rather than with a single causative agent [5,6,26].

Selection of *P. stomatis* was relevant because this species belongs to the family *Pasteurellaceae* and is strongly associated with the oral cavity and upper respiratory tract of domestic dogs and cats [7,27]. In humans, *Pasteurella* spp. are recognized causes of infection after dog and cat bites, scratches, or close animal contact, which can result in serious wound infections [10,28]. The presence of *P. stomatis* in canine periodontal pockets may be clinically relevant, as these sites can act as a reservoir of opportunistic bacteria with potential zoonotic significance. *N. animaloris* was included for similar reasons—it is mainly associated with the respiratory tract and oral surfaces of dogs and cats. Human wound infections caused by *N. animaloris* have been reported after animal bites [12,29,30]. This species remains poorly represented in AMR databases, making all available data valuable, although MIC values cannot yet be confidently translated into clinical susceptibility categories. *S. canis* differs from the Gram-negative species mentioned above because it is a β-haemolytic Lancefield group G streptococcus originally described from animal skin and mucous membranes, especially oral. Human infections are less common but have been reported on multiple occasions [9,31,32]. This is particularly relevant because dogs live in close physical contact with humans, and periodontitis can cause significant chronic pain and discomfort in animals, potentially contributing to pain-related aggression or other unwanted behavior changes [33,34,35]. This issue becomes even more important, considering that modern medicine has expanded the number of people living with immunosuppressive conditions or therapies while animals are increasingly kept as companions [36]. This does not mean that healthy dogs should be viewed as a threat—rather, it reinforces the need to keep animals as healthy as possible and to prioritize preventive veterinary care [16].

In this study, classical bacteriological methods were used to generate preliminary phenotypic susceptibility data and to recover viable isolates, which are essential for MIC testing [17]. MALDI-TOF MS was used for identification, offering a rapid and reliable method for species-level bacterial identification [19]. Nevertheless, this approach also has limitations: it does not detect the entire periodontal pocket microbiome, especially unculturable species; in this study, only three species were selected for further analysis. These findings should be interpreted as describing selected culturable opportunistic bacteria rather than the full microbial complexity of canine periodontitis. The antimicrobial panel used in this study covered several clinically relevant antimicrobial classes: beta-lactams, tetracyclines, folate pathway inhibitors, aminoglycosides, phenicols and fluoroquinolones. This range was useful for comparing MIC distributions across drugs with different mechanisms of action.

The MIC distributions in this analysis differed between bacterial species and antimicrobial agents. *P. stomatis* isolates were generally concentrated at lower MIC ranges for amoxicillin, ciprofloxacin, and chloramphenicol. For *N. animaloris*, MIC values were mostly interpreted descriptively because species-specific breakpoints were unavailable, but the data showed a heterogeneous distribution and several isolates with high MIC values, particularly for doxycycline and trimethoprim–sulfamethoxazole. Among *S. canis* isolates, MIC distributions appeared broader for doxycycline, chloramphenicol, and ciprofloxacin. Overall, this suggests that canine periodontal pockets may contain mixed opportunistic bacteria with variable antimicrobial susceptibility profiles. However, the absence of validated species-specific breakpoints limits interpretation and supports the need for larger datasets. This supports the view that MIC data for these isolates should be interpreted cautiously, because clinically meaningful veterinary breakpoints require integration of MIC distributions, pharmacokinetic parameters, and other factors rather than MIC values alone [14,37].

This study should be interpreted as a pilot investigation rather than as definitive evidence of resistance prevalence or transmission risk. Future studies should include larger dog populations, multiple geographic locations, and molecular detection of resistance genes. Nevertheless, the present data represent a strong first step, as they identify clinically relevant canine oral opportunistic bacteria and provide initial MIC distributions. Overall, these findings highlight the need to integrate veterinary dentistry, the clinical importance of periodontitis, and chronic oral pain in animals into a unified approach to companion-animal health and antimicrobial stewardship.

## 5. Conclusions

This pilot study showed that canine periodontal pockets often harbor culturable opportunistic bacteria, including *Pasteurella stomatis*, *Neisseria animaloris*, and *Streptococcus canis*. As periodontal disease progresses, the abundance of the above-mentioned bacterial species increases; this confirms not only the co-existence of these bacteria within the periodontal microenvironment but also supports the multi-etiological nature of canine periodontal disease. MIC distributions differed between bacterial species and antimicrobial agents, suggesting variable antimicrobial susceptibility profiles. However, interpretation is still limited by the lack of validated species-specific epidemiological cut-off values, since available clinical breakpoints are generally defined at the genus level rather than for these individual species. These findings support the relevance of canine periodontal pockets as a potential reservoir of opportunistic bacteria.

## Figures and Tables

**Table 1:** Clinical characteristics of the 101 dogs included in the study.

Variable		n	%
SEX	Female	51	50.5
	Male	50	49.5
Weight	<5 kg	31	30.7
	5–10 kg	56	55.4
	>10 kg	14	13.9
Age	<2 years	14	13.9
	2–8 years	37	36.6
	>8 years	50	49.5
Diet	Dry	60	59.4
	Mixed	41	40.6

**Table 2:** Number of analyzed bacterial species detected per dog with the classical plating method across periodontal disease stages.

Periodontal Disease Stage	Dogs, n	One Bacterial Species, n (%)	Two Bacterial Species, n (%)	Three Bacterial Species, n (%)
PD1	11	9 (81.8%)	2 (18.2%)	0 (0.0%)
PD2	35	20 (57.1%)	12 (34.3%)	3 (8.6%)
PD3	36	16 (44.4%)	14 (38.9%)	6 (16.7%)
PD4	19	6 (31.6%)	8 (42.1%)	5 (26.3%)
Total	101	51 (50.5%)	36 (35.6%)	14 (13.9%)

**Table 3:** MIC distribution of *Pasteurella stomatis* isolates across tested antimicrobial agents.

Antimicrobial Agent	Concentration of Antimicrobials, mg/L																																			
	0.002	0.003	0.004	0.006	0.008	0.012	0.016	0.023	0.032	0.047	0.064	0.094	0.125	0.19	0.25	0.38	0.50	0.75	1	1.5	2	3	4	6	8	12	16	24	32	48	64	96	128	192	256	>256
Gentamicin											5.6	3.7	3.7	13.0	3.7	9.3	13.0	13.0	5.6	16.7	1.9	1.9	1.9	3.7												3.7
Doxycycline										1.9	5.6	5.6		16.7	20.4	3.7	7.4	11.1	7.4	11.1	1.9					1.9		1.9							3.7	
Trimethoprim—sulfamethoxazole						3.7	5.6	7.4	9.3	14.8	13.0	11.1	14.8	3.7	3.7							7.4							5.6							
Chloramphenicol										3.7				7.4		22.2	11.1	24.1	14.8	5.6	5.6	3.7				1.9										
Amoxicillin							14.8	13.0	5.6	7.4	11.1	14.8	11.1	7.4	3.7	3.7	3.7																		3.7	
Ciprofloxacin	13.0	9.3	18.5	13.0	7.4	3.7	9.3	1.9		1.9			5.6			3.7			1.9	1.9	1.9		3.7						3.7							

The gray-shaded areas represent the concentration intervals tested for each antimicrobial agent. Black vertical bars denote the clinical resistance breakpoints for *Pasteurella* spp. whenever possible, defined by EUCAST.

**Table 4:** MIC distribution of *Neisseria animaloris* isolates across tested antimicrobial agents.

Antimicrobial Agent	Concentration of Antimicrobials, mg/L																																			
	0.002	0.003	0.004	0.006	0.008	0.012	0.016	0.023	0.032	0.047	0.064	0.094	0.125	0.19	0.25	0.38	0.50	0.75	1	1.5	2	3	4	6	8	12	16	24	32	48	64	96	128	192	256	>256
Gentamicin											6.06		3.03	3.03	9.09	3.03	15.2	30.3	12.1	6.06			3.03			3.03	6.06									
Doxycycline													3.03	3.03	6.06	3.03	21.2	6.06	6.06	3.03	9.09	9.09	3.03	3.03	15.2	6.06									3.03	
Trimethoprim—sulfamethoxazole									3.03		3.03	3.03	12.1	6.06	24.2	12.1	15.2		6.06	6.06	3.03								6.06							
Chloramphenicol																	9.09		27.3	15.2	12.1	12.1	6.06	6.06	6.06										6.06	
Amoxicillin								3.03		9.09	12.1		18.2	18.2	12.1	15.2	3.03	6.06																	3.03	
Ciprofloxacin	9.09	15.2	6.06	9.09	6.06		3.03	3.03	3.03	3.03			9.09			3.03	12.1		3.03	9.09				3.03					3.03							

The gray-shaded areas represent the concentration intervals tested for each antimicrobial agent. No species-specific breakpoints or cut-off values are currently available. Red vertical bars denote the clinical resistance breakpoints for *Neisseria gonorrhoeae* defined by EUCAST. The (T)ECOFF values in the table are marked with blue-colored cells.

**Table 5:** MIC distribution of *Streptococcus canis* isolates across tested antimicrobial agents.

Antimicrobial Agent	% of Isolates by Antimicrobial Concentration MIC, mg/L																																				
	0	0.002	0.003	0.004	0.006	0.008	0.012	0.016	0.023	0.032	0.047	0.064	0.094	0.125	0.19	0.25	0.38	0.50	0.75	1	1.5	2	3	4	6	8	12	16	24	32	48	64	96	128	192	256	>256
Gentamicin	2.86								2.86					2.86	2.86		5.71	5.71	5.71	2.86		17.14	8.57	14.29	22.86					2.86						2.86	
Doxycycline												2.9			5.7	5.7	2.9	5.7	5.7	5.7	5.7	2.9	8.6	8.6	11.4	5.7	8.6				2.9					11.4	
Trimethoprim–sulfamethoxazole	5.7								2.9		2.9		17.1	17.1	11.4	5.7	8.6	8.6	2.9	2.9			2.9				5.7			5.7							
Chloramphenicol																		5.7	2.9		2.9	2.9	17.1	8.6	14.3	17.1	20.0									8.6	
Amoxicillin	8.6							5.7		5.7	5.7	2.9	11.4	5.7	17.1	5.7	8.6	8.6		2.9	2.9															8.6	
Ciprofloxacin	2.9	2.9	2.9		5.7							2.9			2.9	2.9	2.9		8.6	8.6	8.6	14.3	8.6	2.9	5.7	2.9	2.9	2.9		8.6							

The gray-shaded areas represent the concentration intervals tested for each antimicrobial agent. Black vertical bars denote the clinical resistance breakpoints defined by EUCAST. Green vertical bars denote the intermediate susceptibility breakpoints established by CLSI. The blue vertical bar represents the breakpoint established by the United States Food and Drug Administration (FDA) for beta-hemolytic *Streptococcus* spp. A value of 0 indicates that there was no bacterial growth detected around the antibiotic strip.

## Data Availability

Data supporting the reported results are available from the corresponding author upon reasonable request.

## Abbreviations

The following abbreviations are used in this manuscript:

MIC	Minimum inhibitory concentration
MALDI-TOF MS	Matrix-assisted laser desorption/ionization time-of-flight mass spectrometry
COHAT	Comprehensive oral health assessment and treatment
PD	Periodontal disease
AML	Amoxicillin
DX	Doxycycline
SXT	Trimethoprim–sulfamethoxazole
CN	Gentamicin
C	Chloramphenicol
CIP	Ciprofloxacin
EUCAST	European Committee on Antimicrobial Susceptibility Testing
CLSI	Clinical and Laboratory Standards Institute
ECOFF	Epidemiological cut-off value
